# Stable nitrogen-doped carbon quantum dots with pH-controlled fluorescence response for Fe^3+^ detection

**DOI:** 10.1038/s41598-026-41900-w

**Published:** 2026-03-03

**Authors:** Rama Juha, Ibrahim Alghoraibi

**Affiliations:** 1 Department of Physics, Faculty of Science, Damascus University, Damascus, Syria; 2 Department of basic and supporting sciences, Faculty of Pharmacy, Arab International University, Ghabaghib, Dara, Syria

**Keywords:** Nitrogen-doped carbon quantum dots (NCQDs), hydrothermal method, fluorescence, fluorescence quenching, CQDs/PVA composite films, quantum yield, HRTEM, Chemistry, Materials science, Nanoscience and technology, Optics and photonics

## Abstract

Nitrogen-doped carbon quantum dots (NCQDs) were synthesized via a facile and optimized hydrothermal method. The as-prepared CQDs were comprehensively characterized using AFM, HRTEM, DLS, ζ-potential, EDX, XRD, Raman, FTIR, UV–Vis, and fluorescence spectroscopy, confirming their nanoscale dimensions, graphitic structure, abundant nitrogen- and oxygen-containing surface functionalities, and excellent aqueous dispersibility. The CQDs exhibited strong blue fluorescence with excitation-independent emission and a relatively high quantum yield of 37.8%. Their fluorescence stability was systematically evaluated over a wide range of pH values, ionic strengths, and solvent environments, demonstrating robust optical performance. Importantly, the fluorescence sensing behavior toward Fe^3+^ ions was critically examined under different pH conditions, revealing that Fe^3+^ detection in alkaline media is severely hindered by Fe(OH)_3_ precipitation, which leads to misleading quenching effects. By conducting sensing experiments under strongly acidic conditions (pH = 2), where Fe^3+^ remains fully soluble, a clear and reliable fluorescence quenching response was achieved over a wide linear range (20–1000 µM), with a detection limit of 10.25 µM. In addition, the practical applicability of the CQDs was demonstrated through fluorescent ink and CQDs/PVA composite films.

## Introduction

Carbon quantum dots (CQDs) have emerged as a promising class of carbon-based nanomaterials owing to their unique optical properties, including strong fluorescence, tunable emission, excellent water dispersibility, and low toxicity^1^. These features, combined with their facile synthesis from inexpensive precursors, have enabled their widespread application in sensing, bioimaging, optoelectronics, and security-related technologies^2–4^. In particular, heteroatom doping has proven to be an effective strategy to further enhance the optical performance of CQDs by modulating their electronic structure and surface chemistry^5^. Among various dopants, nitrogen is especially attractive due to its comparable atomic size to carbon and its ability to introduce additional emissive states, improve quantum yield, and enhance surface passivation^6^. Nitrogen-doped carbon quantum dots (N-CQDs) are typically synthesized via hydrothermal or solvothermal routes using small organic molecules^7,8^. Among the different synthetic routes reported for the preparation of CQDs, the hydrothermal approach has evolved as one of the most reliable and widely adopted methods due to its simplicity, versatility, and high controllability. The method allows for progressive carbonization and controlled nucleation under moderate temperature and pressure in a closed aqueous environment, yielding CQDs with narrow size distribution, rich surface functional groups, and excellent colloidal stability. In contrast to microwave-assisted synthesis, which features rapid reaction rates but often shows low controllability for the carbonization step or batch-to-batch reproductions, the hydrothermal process provides better homogeneity and optical property controllability^4^. Compared with other more complex hydro-processing methods, the hydrothermal approach is more straightforward, environmentally friendly, and scalable, making it favorable for producing high-quality CQDs for applications such as sensing, bioimaging, and optoelectronic devices. The optical properties of the resulting nanomaterials are highly sensitive to synthesis parameters, including reaction time, precursor ratio, and dopant concentration, as these factors directly influence the degree of carbonization, particle size, and surface functionalization^9^. Consequently, systematic optimization of synthesis conditions is essential to achieve CQDs with high fluorescence efficiency and structural stability. Despite extensive research efforts, significant discrepancies in reported optical performance and quantum yield values persist, highlighting the need for well-controlled synthesis and comprehensive characterization^10,11^. Beyond their intrinsic photophysical properties, CQDs have attracted considerable attention as fluorescent probes for metal-ion sensing, particularly Fe^3+^ ions, due to their environmental and biological relevance. Most reported CQD-based Fe^3+^ sensors operate under neutral or alkaline conditions, where fluorescence quenching is attributed to strong coordination between Fe^3+^ ions and surface functional groups. However, under alkaline conditions, Fe^3+^ readily undergoes hydrolysis and precipitates as Fe(OH)_3_, reducing the concentration of free Fe^3+^ ions and complicating the interpretation of sensing mechanisms. Consequently, interpreting the interaction between CQDs and Fe^3+^ ions in alkaline media may be challenging. In contrast, sensing under acidic conditions preserves Fe^3+^ ions in their soluble form, yet this regime has received limited attention due to concerns regarding fluorescence instability at low pH In this context, the present study does not merely report a CQD-based Fe^3+^ sensor, but systematically investigates the influence of solution pH on the reliability and interpretability of fluorescence quenching. By explicitly comparing alkaline and strongly acidic environments, this work clarifies the critical role of Fe^3+^ speciation and hydrolysis in CQD-based sensing systems and identifies conditions under which chemically meaningful detection can be achieved. In this work, nitrogen-doped CQDs were synthesized from citric acid and urea via an optimized hydrothermal method. The effects of reaction time and urea content on the optical properties were carefully investigated to establish optimal synthesis parameters. Comprehensive structural and spectroscopic analyses confirmed the successful formation of well-dispersed N-CQDs with stable fluorescence characteristics and abundant surface functional groups suitable for metal-ion interaction. Methanol-assisted purification was employed as an effective post-synthesis treatment to remove aggregated by-products, resulting in improved colloidal stability and optical reproducibility. Importantly, the fluorescence sensing behavior toward Fe^3+^ ions was systematically evaluated under strongly acidic conditions, ensuring that Fe^3+^ remains soluble and chemically available. This approach enables a more reliable interpretation of the quenching mechanism and provides practical guidance for designing CQD-based sensing systems in environments where metal-ion speciation is strongly pH-dependent. In addition, the synthesized N-CQDs exhibited excellent fluorescence stability over a wide pH range and in high ionic strength environments. Furthermore, their practical applicability was demonstrated through successful utilization as a fluorescent ink and as CQDs/PVA composite films, highlighting their potential for optical and anti-counterfeiting applications.

## Results and discussion

### Structural and chemical characterization of CQDs

 To comprehensively evaluate the morphology, crystallinity, and surface chemistry of the produced carbon quantum dots. These techniques include AFM, HR-TEM, DLS, ζ-potential measurement, EDX, XRD, Raman, and FTIR analyses.

**Fig. 1:**
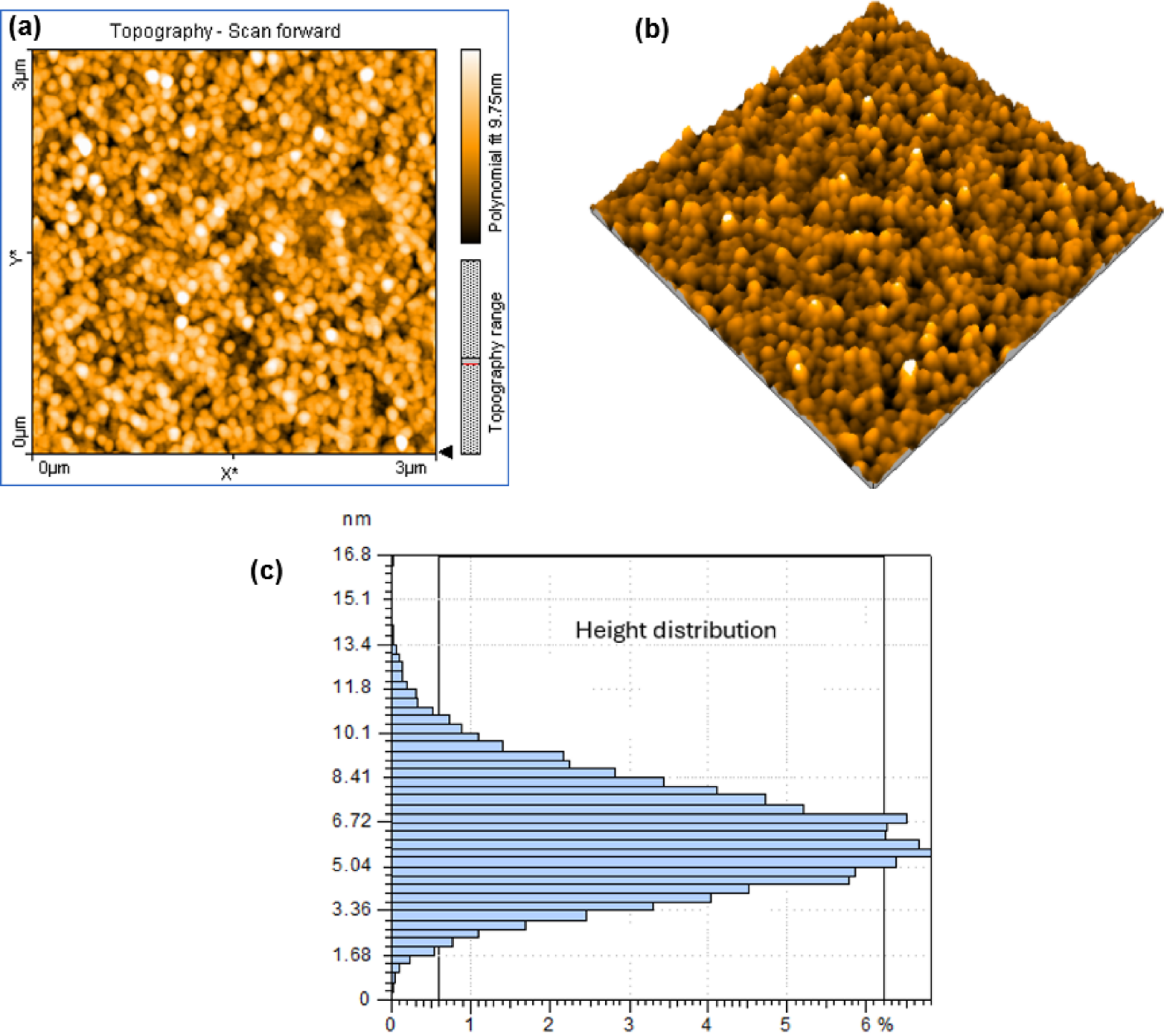

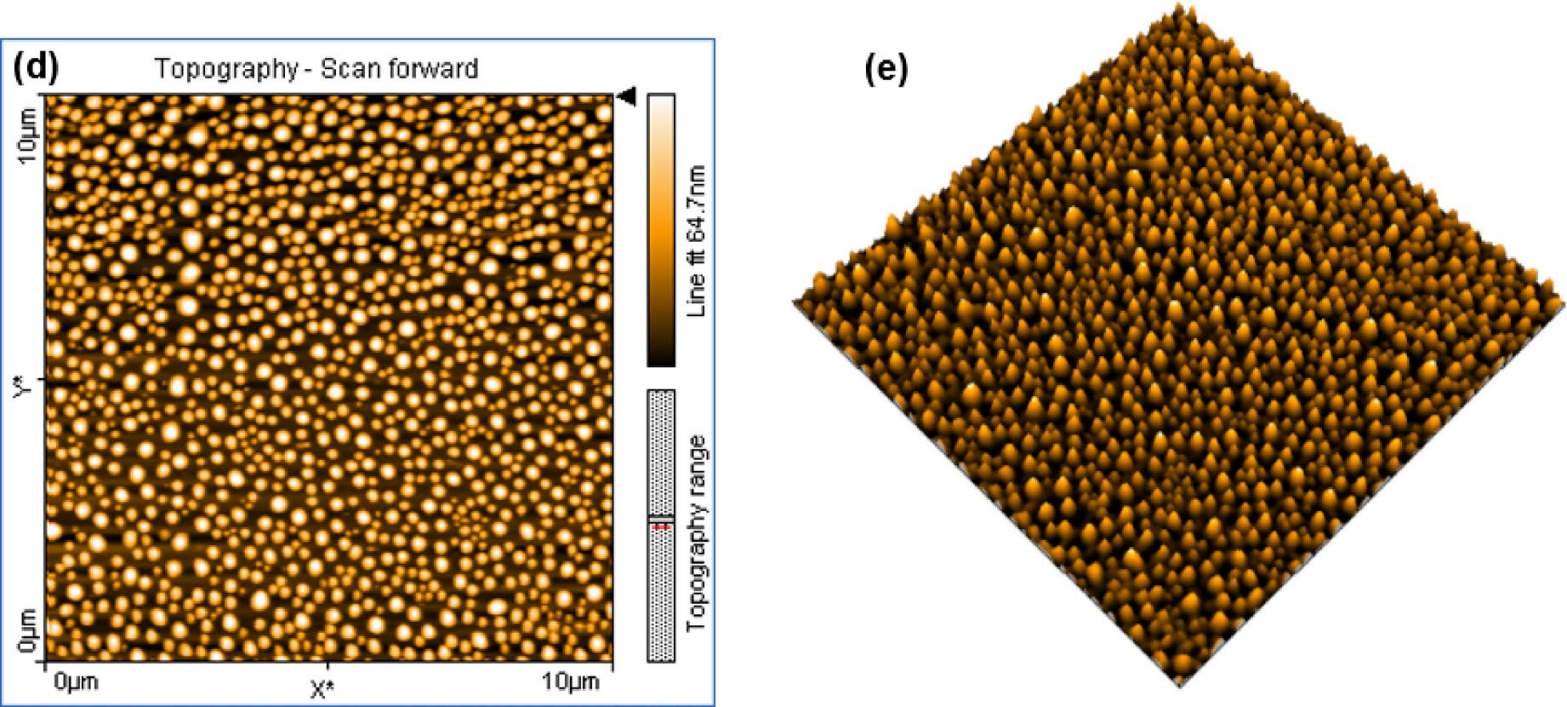
AFM characterization of CQDs: (**a**, **b**) 2D and 3D topographic images before thermal treatment; (**c**) height distribution; (**d**, **e**) corresponding 2D and 3D images after thermal treatment.

The surface morphology of the synthesized carbon CQDs was examined using atomic force microscopy (AFM). The 2D topographic image over a 3 μm × 3 μm area (Fig. 1a) revealed a dense and uniform distribution of quasi-spherical nanoparticles without any signs of aggregation. The corresponding 3D image (Fig. 1b) further confirms a homogeneous surface with nanoscale roughness. Quantitative analysis showed low roughness parameters (Sa = 1.6 nm and Sq = 2.0 nm), reflecting a smooth surface and well-dispersed CQDs. Height distribution analysis (Fig. 1c) indicates a mean particle height of approximately 5.9 nm, consistent with the formation of ultra-small, uniformly distributed CQDs. To enhance particle visibility and promote a more uniform distribution across the substrate, the deposited CQDs underwent a brief thermal treatment prior to AFM imaging. The post-treated sample exhibited improved particle contrast and sharper morphological features in both 2D and 3D AFM images as shown in Fig. 1(d and e respectively). Quantitative analysis of the thermally treated sample indicates that the total surface area occupied by CQDs is about 60.1%, with a particle density of 28 grains/µm^2^. Surface roughness parameters (Sa = 14 nm and Sq = 17 nm) reveal increased nanoscale roughness. The thermal treatment thus facilitated clearer imaging while maintaining the nanoscale features of the CQDs.

**Fig. 2:**
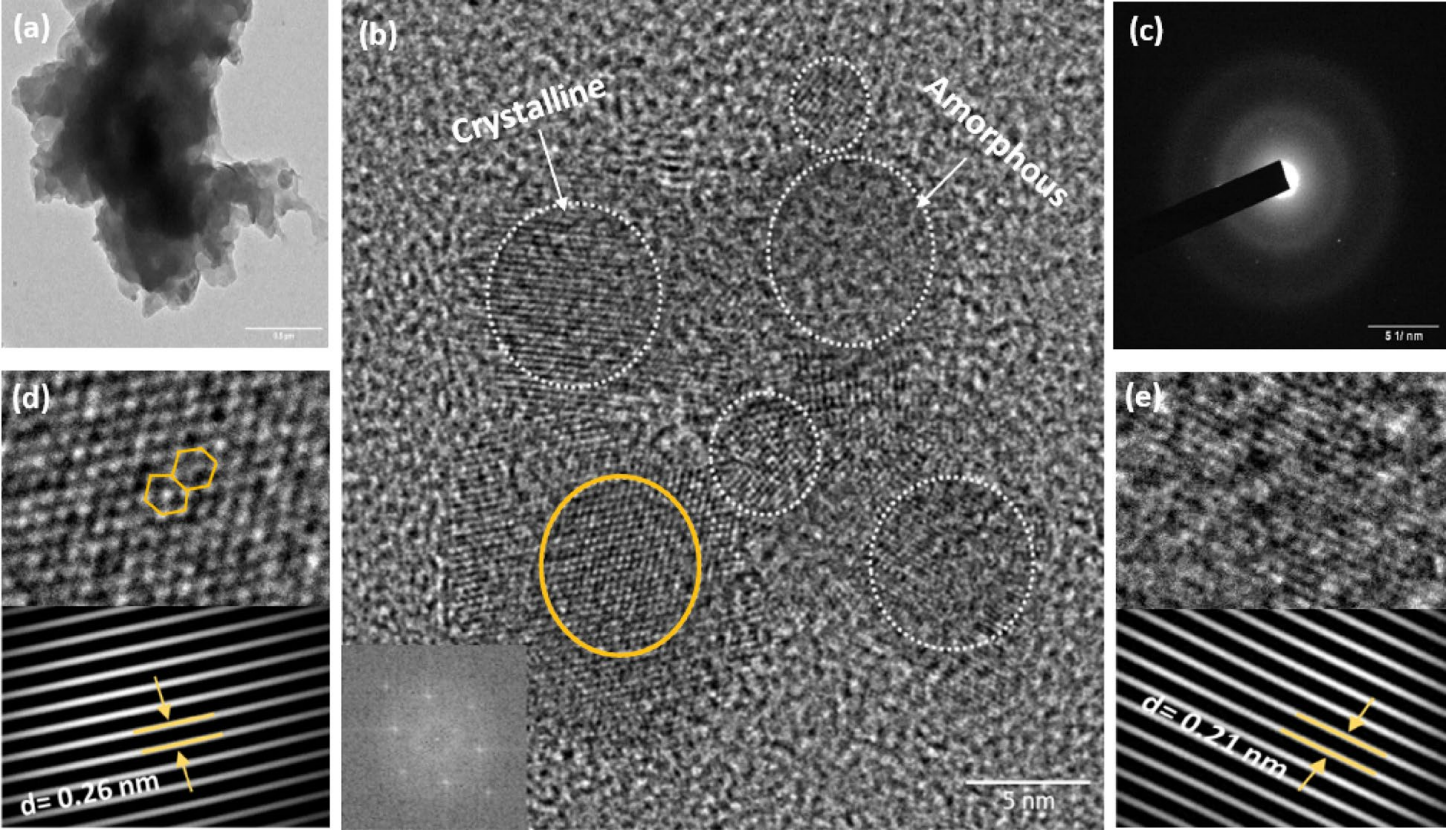
TEM and HRTEM characterization of the synthesized CQDs: (**a**) Low-magnification TEM image showing the overall morphology; (**b**) HRTEM image revealing crystalline and amorphous regions with FFT inset (yellow circle); (**c**) SAED pattern exhibiting diffuse rings characteristic of partially ordered graphitic domains; (**d**,** e**) Lattice-resolved HRTEM images showing interplanar spacings of 0.26 nm and 0.21 nm, corresponding to the (100) plane of graphitic carbon.

To further investigate the internal structure and crystallinity of the CQDs beyond the surface morphology observed by AFM, high-resolution transmission electron microscopy (HRTEM) was performed. The HRTEM image (Fig. 2b) revealed that the CQDs have an average diameter of approximately 5.6 nm, consisting of graphitic nanocrystalline domains embedded within an amorphous carbon matrix. The fast Fourier transform (FFT) of the selected area (yellow circle), shown in the inset of Fig. 2, indicates hexagonal symmetry characteristic of graphitic ordering. This observation was further supported by the selected area electron diffraction (SAED) pattern presented in Fig. 2c, which shows diffuse ring patterns, reflecting the degree of structural order in the CQDs, an important factor influencing their optical properties. In addition, the CQDs have clear lattice fringes with d- spacings of 0.26 nm, 0.21 nm, which can be assigned to the (100) and (101) lattice planes of graphitic carbon^12,13^, as shown in (Fig. 2d and e). However, the absence of the 0.33 nm graphitic (002) spacing suggests limited interlayer stacking and turbostratic disorder, likely influenced by surface functionalization and lattice distortion at the nanoscale^14^.

**Fig. 3:**
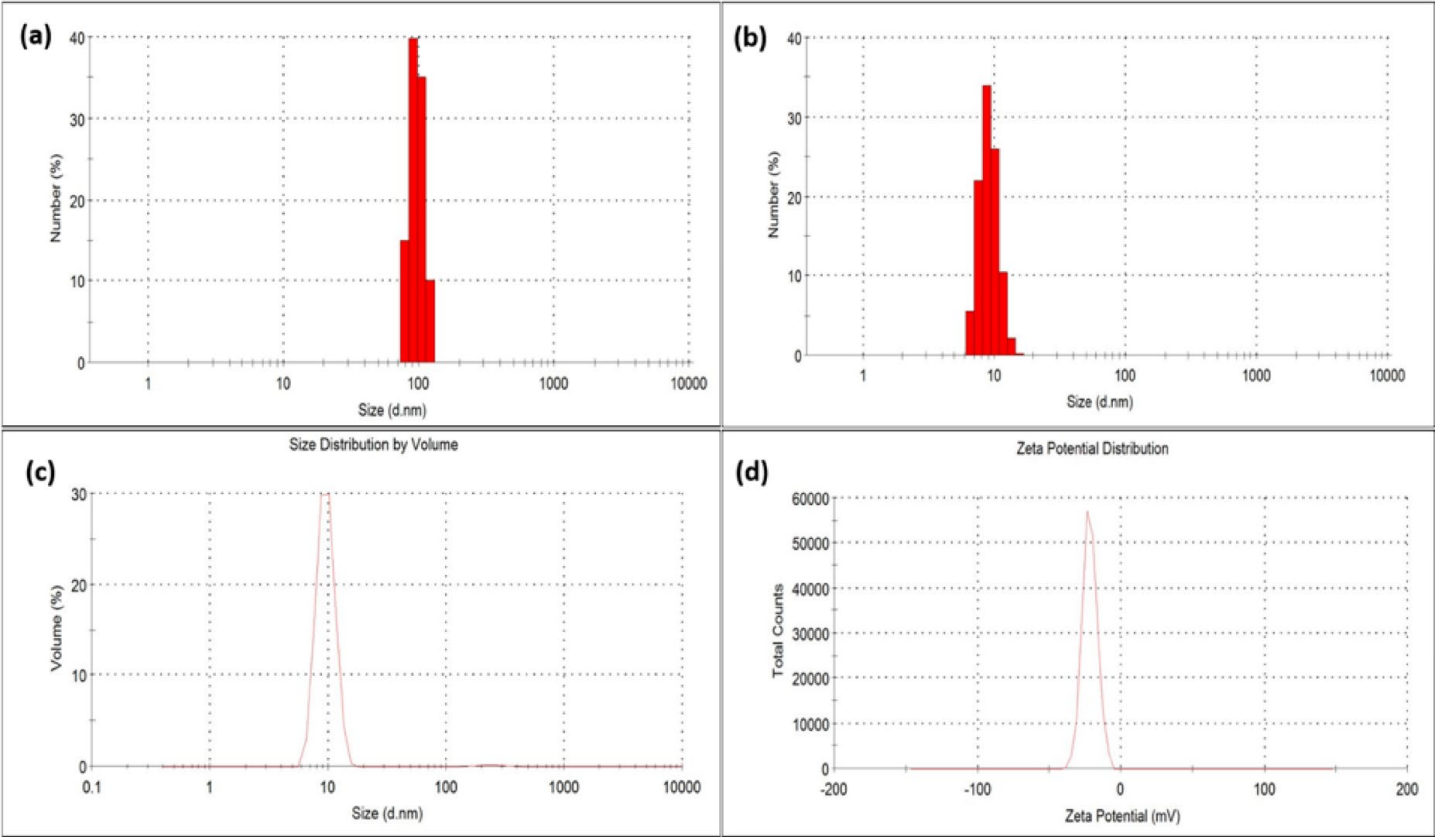
DLS number CQDs size distributions before (**a**) and after methanol purification (**b**,** c**), showing removal of aggregates and a narrow peak at ~ 9.6 nm; (**d**) zeta potential curve indicating a negative surface charge of − 22.3 mV.

Dynamic light scattering (DLS) measurements were carried out to determine the CQDs average hydrodynamic size number distribution. In addition, zeta potential (ζ) analysis was performed to evaluate the surface charge and electrostatic repulsion responsible for the colloidal stability of the dispersion. DLS analysis of the CQDs dispersion prior to methanol purification revealed a sharp number-weighted peak at ~ 100 nm, which can be attributed to the presence of aggregates (Fig. 3a). This observation agrees with the number-based nature of DLS, where even a small fraction of larger particles can dominate the scattering response and distort the apparent size number distribution^15^. After methanol purification, the size number distribution changed significantly: the number-weighted plot exhibited a distinct peak at ~ 9.6 nm, and the volume-weighted representation confirmed that the majority of particles were concentrated within this range (Fig. 3b, c). These findings indicate that methanol purification effectively removes aggregated or polymeric by-products, yielding well-dispersed CQDs. Furthermore, it is observed that the hydrodynamic diameter of CQDs in aqueous dispersion was larger than the size measured by HRTEM under high-vacuum, dry conditions. This discrepancy is generally attributed to the presence of surface-bound functional groups^16^. The ζ-potential of − 22.3 mV (Fig. 3d) confirms that the CQDs possess a negative surface charge. This negative potential arises from surface functional groups that ionize in aqueous medium, resulting in a negatively charged CQD surface that contributes to colloidal stability^17^.

**Fig. 4:**
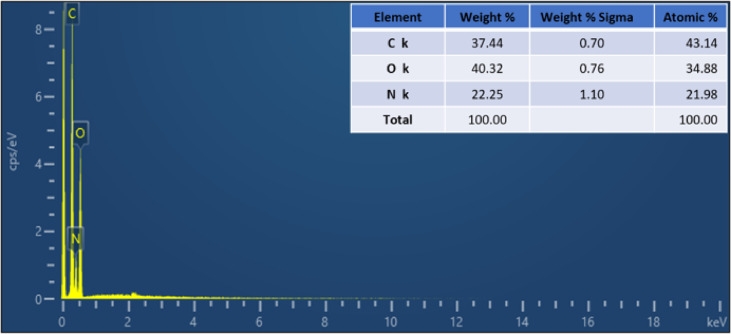
EDX spectrum of the CQDs with inset elemental composition.

Following the morphological and dimension analyses, the elemental composition of the CQDs was examined by EDX. The spectrum (Fig. 4) displayed distinct signals for carbon, oxygen, and nitrogen only, with any metallic impurities, confirming the high purity of the synthesized CQDs. Elemental quantification showed atomic percentages of 43.14% C, 34.88% O, and 21.98% N. The relatively high nitrogen and oxygen contents further confirm the heteroatom-rich nature of the CQDs.

**Fig. 5:**
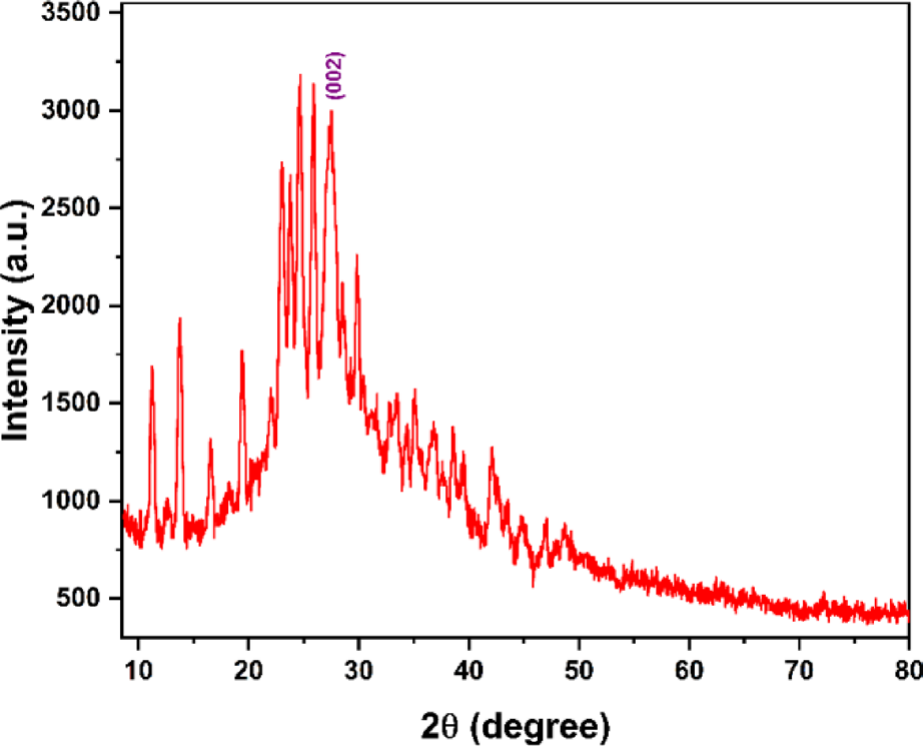
XRD pattern of CQDs showing mixed amorphous and nanocrystalline graphitic phases.

Subsequently, X-ray diffraction (XRD) analysis was performed to evaluate the structural ordering of the synthesized CQDs (Fig. 5). The diffraction pattern exhibits several sharp features superimposed on a broad background, suggesting localized structural ordering within an overall disordered carbon framework. Such a structural profile is characteristic of carbon nanostructures composed of nanocrystalline graphitic domains embedded in an amorphous matrix. Particular attention was given to the diffraction feature centered at 2θ ≈ 27.42°, which can be indexed to the (002) plane of graphitic carbon^18^. Using Bragg’s law (Eq. 1), the corresponding interlayer spacing was calculated to be d = 0.325 nm, which is close to the characteristic (002) spacing of turbostratic or nanocrystalline graphite. This observation supports the presence of localized graphitic domains within the CQDs. Moreover, this result is consistent with the HRTEM and SAED analyses, which revealed lattice fringes and diffraction features indicative of nanoscale graphitic ordering with limited long-range crystallinity.

1 $$d = n\lambda /(2{\text{ }}\sin \;\theta )$$

Where λ is the wavelength of incident X-rays (λ = 1.54060 Å), and θ is the position of the related plane peak, n is a positive integer (*n* = 1).

**Fig. 6:**
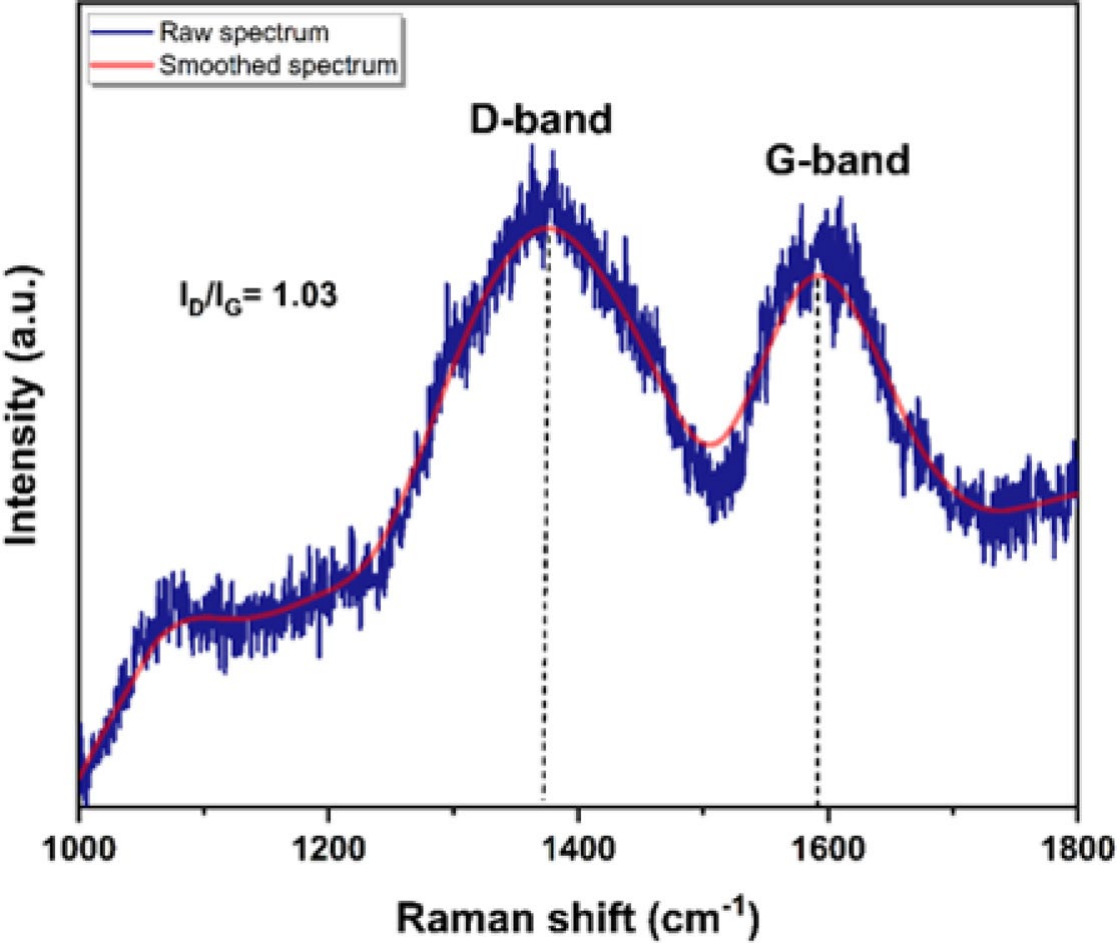
Raman spectrum of the CQDs. The raw data (blue) are shown together with a smoothed curve (red) for visual clarity.

Raman spectroscopy was employed to further investigate the structural order and defect density of the CQDs. The resulting spectrum displayed two characteristic bands: a D band at 1375 cm^−1^ and a G band at 1591 cm^−1^ (Fig. 6). The D band is usually associated with disorder-induced vibrations of carbon atoms in defective or sp^3^-hybridized domains, while the G band corresponds to the in-plane E_2_g vibrational mode of sp^2^-hybridized carbon atoms in graphitic structures^19,20^. The intensity ratio (I_D_/I_G_) was determined to be 1.03, which suggests a balance between ordered sp^2^ graphitic domains and disordered sp^3^ defect sites in the CQD structure. Notably, the presence of nitrogen can introduce structural distortions, where the incorporation of nitrogen atoms leads to lattice defects and bond rehybridization, resulting in an increased contribution of sp^3^-hybridized carbon associated with C–N and C–O bonding. Such nitrogen-related defects are considered important because they modulate the electronic structure and enhance the surface reactivity of NCQDs. This interpretation is consistent with prior reports on NCQDs obtained from different carbon/nitrogen precursors (e.g., glucose–NH_3_), where nitrogen doping was shown to promote sp^3^ bonding at the expense of sp^2^ domains while preserving a graphitic core^20^. In this context, the relatively high I_D_/I_G_ ratio further supports the presence of heteroatom-induced structural disorder, which is commonly observed in nitrogen-doped carbon quantum dots and is known to play a key role in tuning their electronic properties and enhancing surface-related functionalities.

**Fig. 7:**
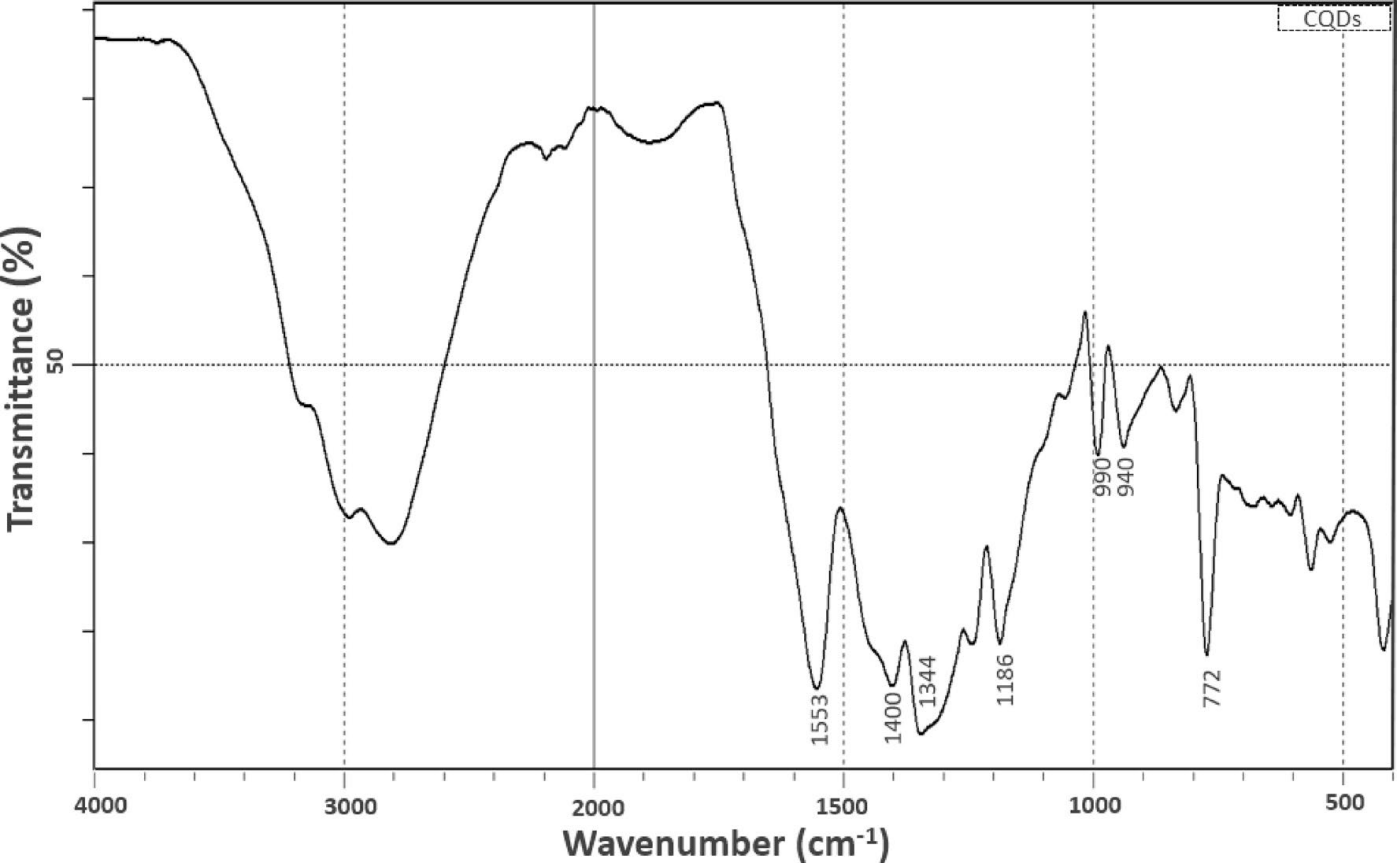
FTIR spectrum of the synthesized CQDs.

To gain deeper insights into the surface chemistry of the synthesized CQDs, FTIR spectroscopy was employed to identify the functional groups present on their surface (Fig. 7). Several characteristic absorption bands were observed within the 4000–400 cm^−1^ region. A broad band in the range of 2500–3400 cm^−1^ was assigned to overlapping O–H and N– H stretching vibrations^21,22^, indicating the presence of hydroxyl and amine groups that render the CQDs hydrophilic and enhance their stability and dispersibility in aqueous media. Weak absorptions at 1710 and 1645 cm^−1^ were attributed to C = O stretching vibrations^22,23^, where the band at 1710 cm^−1^ is associated with carboxyl groups, while the absorption at 1645 cm^−1^ can be related to amide I vibrations and/or conjugated C = C stretching. The very low intensity of these bands suggests that condensation or cyclization processes occurred during the hydrothermal synthesis. The band at 1553 cm^−1^ corresponds to N–H bending^24^, possibly related to amide or amine groups, while the peaks at 1400 and 1344 cm^−1^ are ascribed to C–N stretching vibrations^21,24^, further supporting the presence of nitrogen-containing functionalities. Moreover, the absorption at 1186 cm^−1^ was assigned to C–O stretching^21^, confirming the presence of oxygenated groups on the CQD surface. Finally, the bands at 772, 940, and 990 cm^−1^ are associated with aromatic C–H out-of-plane bending vibrations^25–27^. Collectively, these surface functional groups confirm the successful incorporation of nitrogen and oxygen heteroatoms, which play a crucial role in enhancing the optical properties of the CQDs and thereby broaden their potential applications in bioimaging, sensing, and catalysis. Overall, the combined morphological, structural, and chemical analyses consistently confirm that the synthesized CQDs possess nanoscale dimensions, graphitic crystallinity, and abundant nitrogen- and oxygen-containing surface functionalities, which collectively contribute to their excellent aqueous dispersibility and strong fluorescence behavior.

### Optical properties

**Fig. 8:**
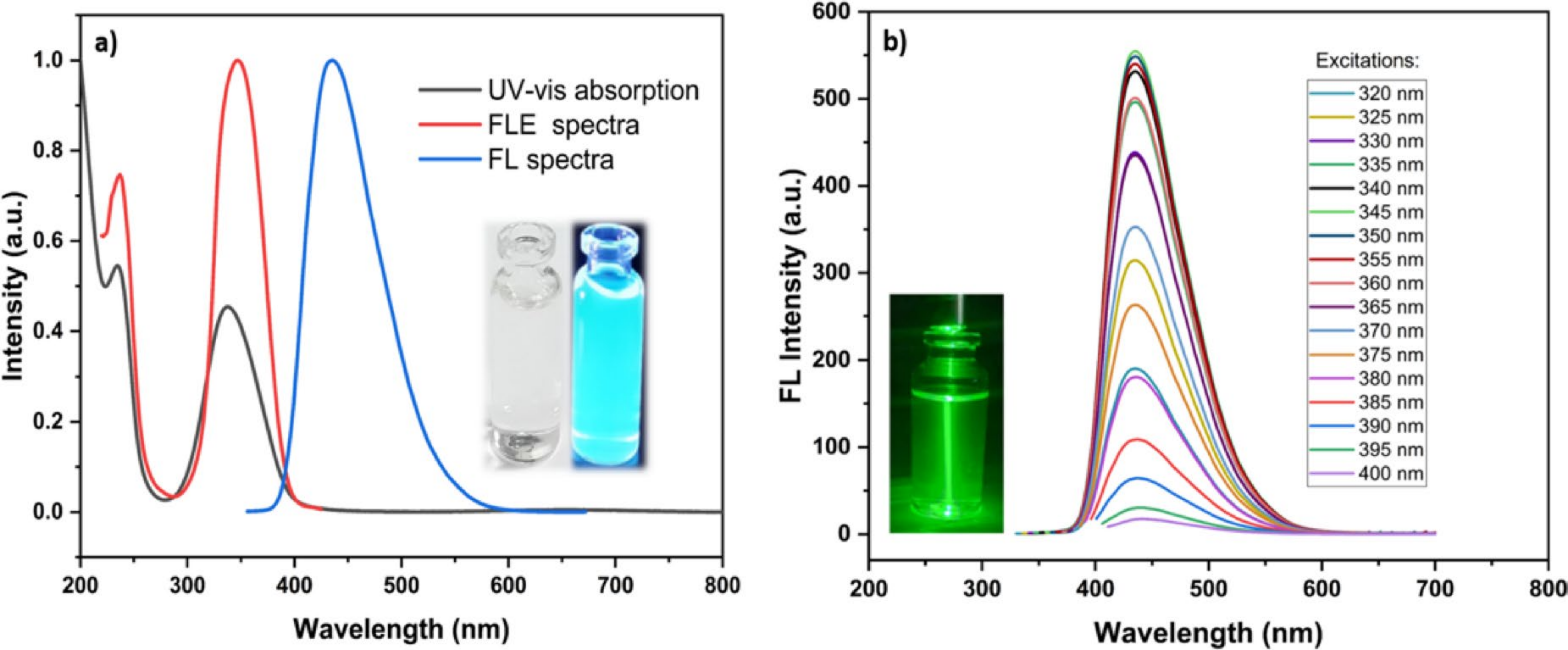
(**a**) UV–vis absorption, fluorescence excitation (FLE), and fluorescence emission (FL) spectra of the synthesized CQDs; the inset shows optical photographs of the aqueous CQDs dispersion under visible light (left) and 365 nm UV illumination (right), (**b**) Fluorescence emission spectra recorded at different excitation wavelengths (320–400 nm), highlighting the excitation-independent emission behavior, the inset photograph shows the Tyndall effect in the CQDs dispersion.

**Fig. 9:**
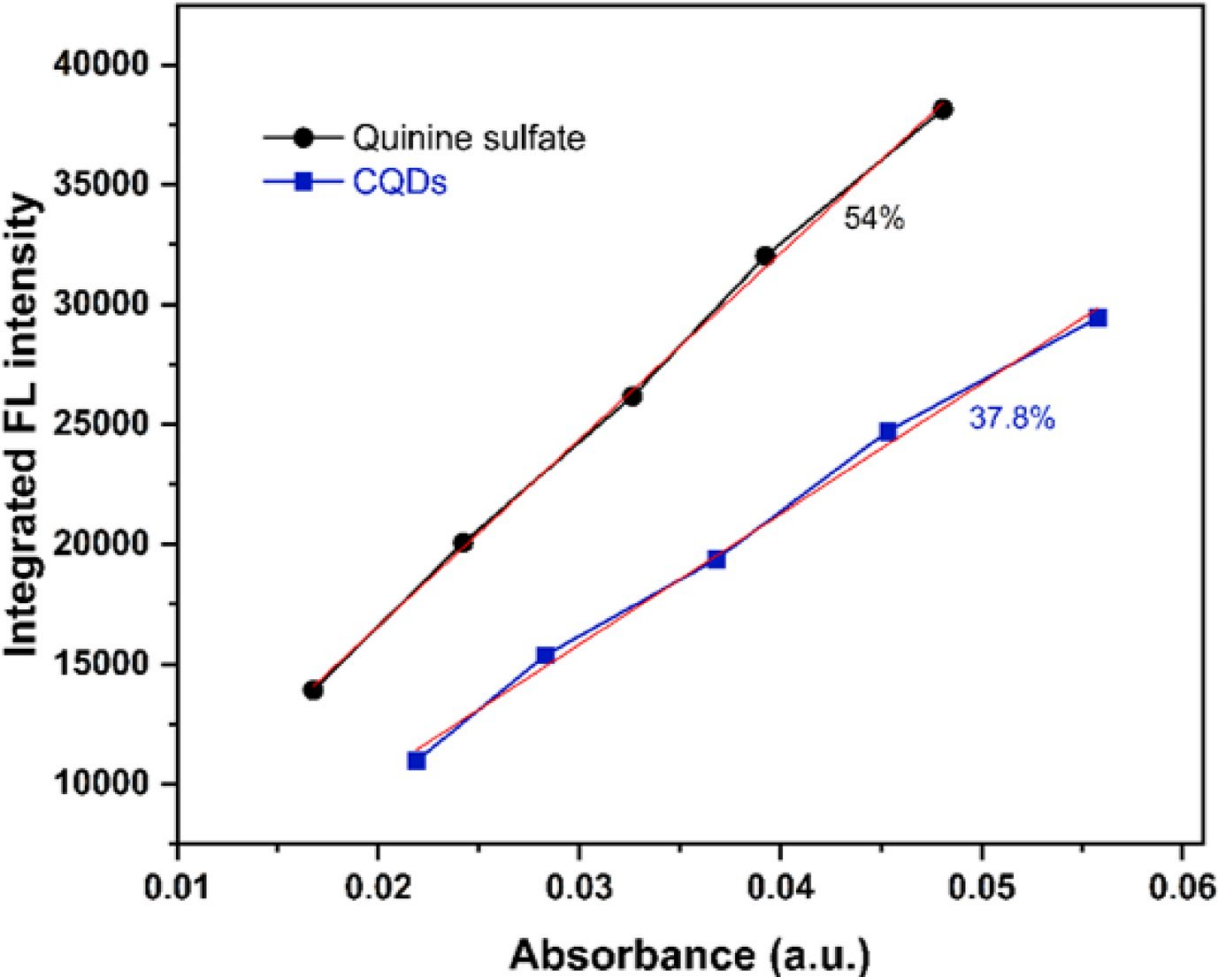
Determination of CQDs quantum yield using the relative slope method with quinine sulfate as the reference standard.

The fluorescence properties of the newly synthesized CQDs are depicted in (Fig. 8). In the UV–vis absorption spectrum of the CQDs aqueous solution (Fig. 1a), two distinct optical absorption bands are observed at 235 nm and 338 nm. The absorption band at 235 nm is attributed to the characteristic π ─ π* transition of C = C (sp^2^) domains, while the band at 338 nm corresponds to n ─ π* transition associated with surface C = O and other heteroatom-containing functional^11,28^. The CQDs exhibit optimal excitation and emission wavelengths at 347 nm and 435 nm, respectively. Under UV lamp illumination (λₑₓ = 365 nm), the aqueous dispersion emits a bright blue fluorescence (inset of Fig. 8a), consistent with the emission behavior shown in (Fig. 8b), where the emission intensity initially increases with excitation wavelength up to 345 nm and then gradually decreases with further increase. Despite the variation in excitation wavelength, no shift of the emission peak was observed, remaining fixed at 435 nm, which indicates excitation-independent emission in the 320–400 nm range. This behavior is attributed to a relatively uniform particle size distribution and a dominant emissive surface state of the CQDs^29^. Moreover, the presence of surface amino groups effectively passivates surface trap states, reducing non-radiative recombination pathways and resulting in a dominant radiative emissive transition^16,30^. The inset photograph in (Fig. 8b) demonstrates the Tyndall effect, indicating the colloidal nature of the synthesized CQDs. When a green laser beam (532 nm) passed through the aqueous CQDs dispersion, a distinct light path was clearly visible due to scattering by the nanosized particles, whereas no beam was observed in pure water. This observation confirms the good colloidal dispersion and nanoscale size of the CQDs in solution. The quantum yield (QY) of the CQDs was calculated to be approximately 37.8% (Fig. 9).

**Fig. 10:**
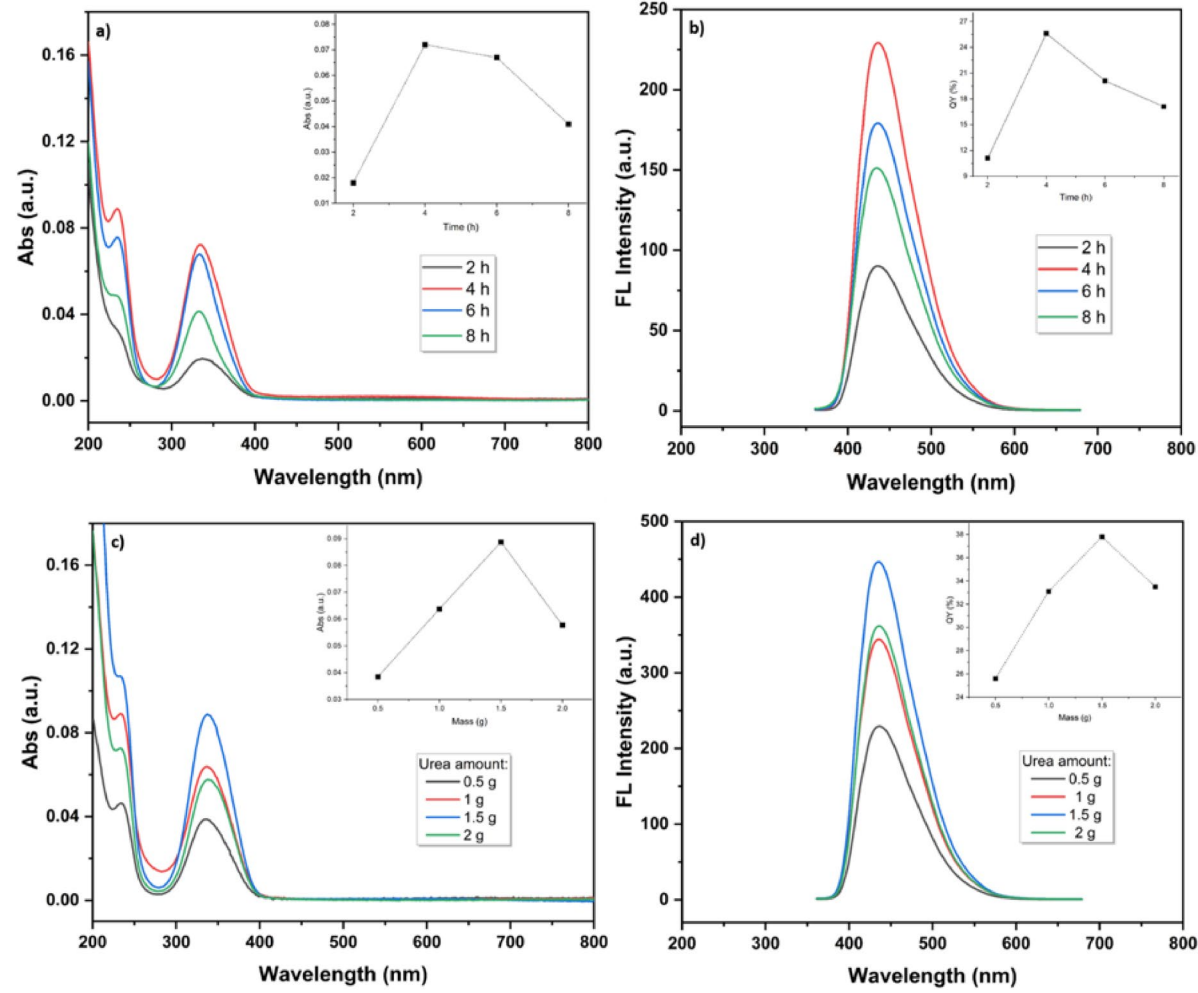
UV–vis and fluorescence spectra of NCDs showing the effects of reaction time (**a**, **b**) and urea mass (**c**, **d**) on their optical properties. Insets: absorbance and quantum-yield variations under the same conditions.

To determine the optimal synthesis conditions, the effect of reaction time on the optical properties of the CQDs was investigated while keeping the citric acid mass (1 g), urea mass (0.5 g), reaction temperature (180 °C), and water volume (25 mL) constant. UV–Vis absorption and fluorescence spectra were recorded for samples synthesized at different reaction times (2, 4, 6, and 8 h). As shown in Fig. 10a, b, the sample prepared at 4 h exhibited the highest absorption and fluorescence intensities. In contrast, longer reaction times (6 and 8 h) resulted in a noticeable decrease in fluorescence intensity, a behavior likely attributed to further carbonization and subtle changes in surface chemical states, which may collectively reduce the overall fluorescence efficiency. QY measurements further confirmed that 4 h is the optimal reaction time for achieving the highest fluorescence performance. Therefore, a reaction time of 4 h was selected for all subsequent experiments. Similarly, the effect of urea amount on the optical properties of the NQCDs was investigated by varying the urea mass (0.5, 1, 1.5, and 2 g). As shown in Fig. 10c, d, the sample synthesized with 1.5 g of urea exhibited the highest absorption and fluorescence intensities, as well as the highest quantum yield. Based on these results, 1.5 g of urea was selected for preparing the optimal CQDs sample. Under the optimized synthesis conditions (180 °C, 4 h reaction time, and 1.5 g urea), the hydrothermal process afforded the CQDs with an isolated yield of approximately 15% after purification. To assess batch-to-batch reproducibility, three independent syntheses were performed under identical conditions. The obtained CQDs exhibited highly consistent fluorescence performance, with an average quantum yield of 36.8 ± 0.9% (*n* = 3), demonstrating good reproducibility of the synthetic procedure.

**Fig. 11:**
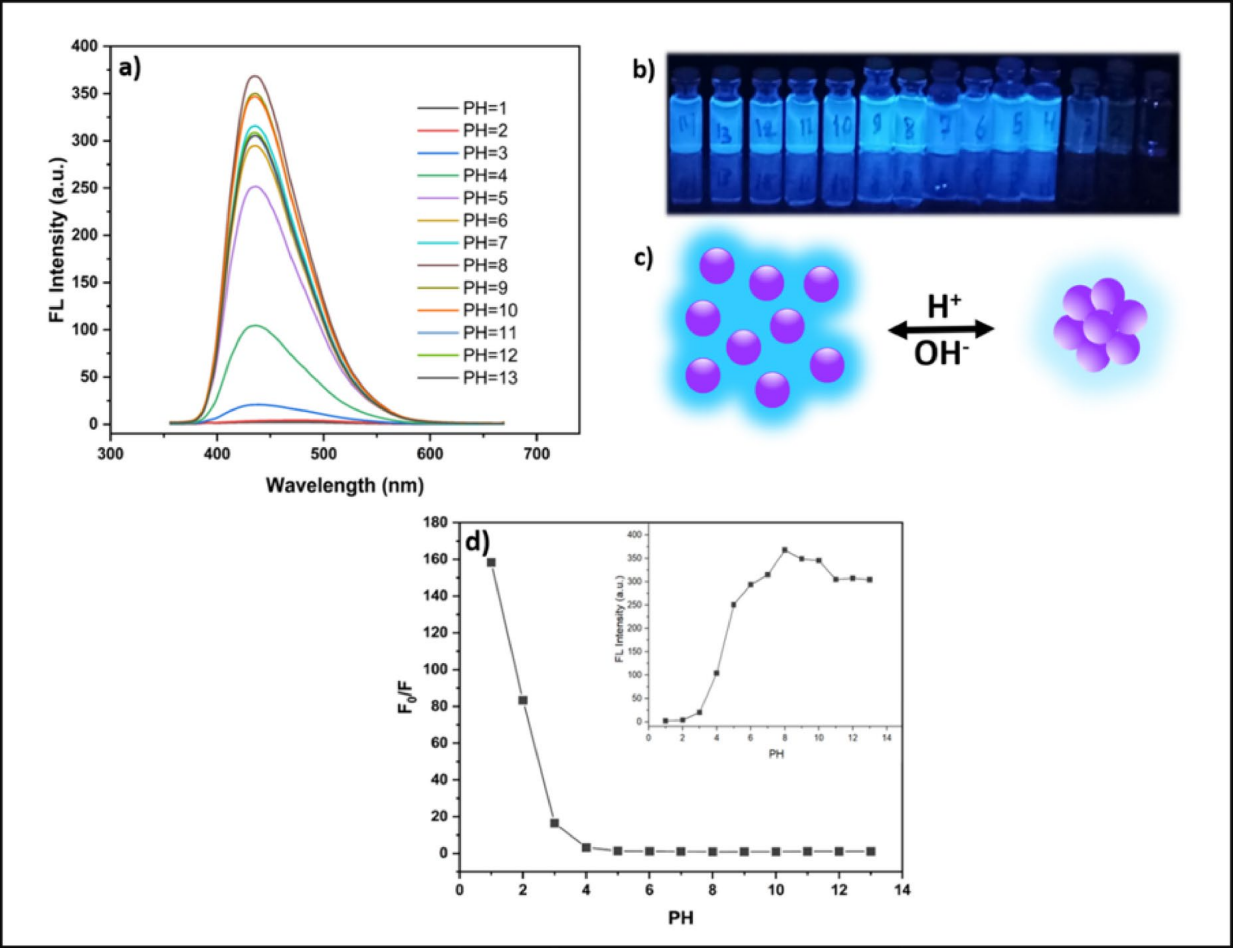
Effect of pH on CQD fluorescence: (**a**) FL spectra, (**b**) photographs under 365 nm UV light, (**c**) schematic illustration of protonation–deprotonation effects on particle dispersion, and (**d**) F_0_/F variation with pH, with an inset showing emission intensity versus pH.

The stability of CQDs across a broad pH range is a critical parameter for sensing applications. Accordingly, the fluorescence (FL) intensity of the synthesized CQDs was systematically measured in aqueous solutions with varying pH values (Fig. 11a, b). The FL response exhibited a clear pH dependence: the emission intensity was strongly quenched under highly acidic conditions (pH 1–3), increased gradually with rising pH, and reached a maximum at approximately pH 8, followed by a slight decrease in the alkaline range. This behavior can be attributed to the protonation/deprotonation of surface functional groups. In acidic media, protonation of the functional groups reduces the surface charge, promotes aggregation, and introduces non-radiative trap states, resulting in severe quenching. In contrast, deprotonation near neutral to mildly alkaline pH enhances negative surface charge, improves colloidal stability, and passivates trap states, thereby maximizing emission intensity, this concept is illustrated schematically in Fig. 11c. To highlight the strong quenching at low pH values, the fluorescence intensity ratio F_0_/F was plotted against pH (Fig. 11d). The exceptionally high F_0_/F values at pH 1–2 confirm strong fluorescence quenching, whereas values close to unity at pH ≥ 4 indicate stable emission over a wide neutral-to-alkaline range. Notably, no shift in the emission peak was observed across the investigated pH range, demonstrating that the emissive state of the CQDs remained stable. Such spectral stability is advantageous for intensity-based sensing applications, as it ensures consistent emission color and reliable signal readout. These results are consistent with previous studies. Huang et al., reported that the fluorescence of carbon dots increased with pH and peaked around pH 8 before decreasing slightly at higher values^31^. Similarly, Chang et al., showed that CQDs displayed maximum emission near neutral pH, with diminished intensities under strongly acidic and alkaline conditions^32^. In contrast, Guo et al. observed an opposite trend, where strong emission was detected at acidic pH (2–4), followed by gradual quenching toward alkaline values^33^. Zamora-Valencia et al. also described enhanced fluorescence in acidic media with reduced intensity in basic environments^34^. Such discrepancies highlight the crucial role of precursor composition, doping, and surface chemistry in determining the pH-dependent fluorescence of CQDs.

**Fig. 12:**
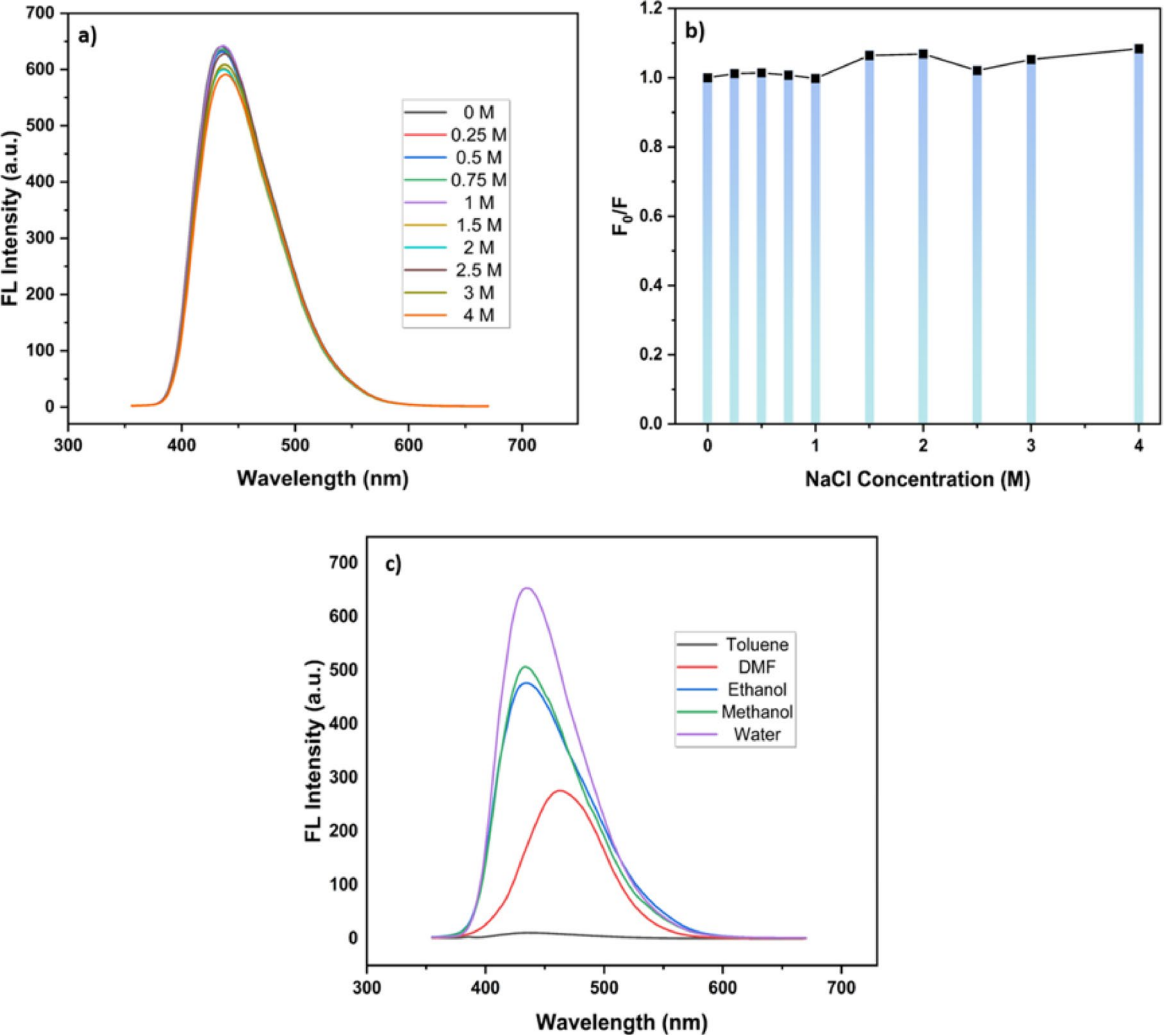
Effect of ionic strength and solvent environment on the fluorescence of CQDs: (**a**) emission spectra and (**b**) relative fluorescence intensity (F_0_/F) under different NaCl concentrations, and (**c**) fluorescence spectra of CQDs dispersed in different solvents, showing the highest emission intensity in water.

Similarly, the influence of ionic strength on the photoluminescence of the synthesized CQDs was systematically examined by monitoring the fluorescence of the CQDs under increasing NaCl concentrations (0–4 M) (Fig. 12a, b). The emission spectra revealed that the peak position remained essentially constant, with only slight variations in intensity across the studied range. Correspondingly, the calculated F0​/F values stayed close to unity, indicating negligible quenching and confirming the excellent salt tolerance of the CQDs. Notably, while most reported studies have limited their evaluation of NaCl effects to concentrations up to ~ 2 M^21,35,36^, our results demonstrate sustained fluorescence stability up to 4 M. This extended salt tolerance underscores the exceptional stability of the synthesized nanomaterials and broadens their applicability in high-salinity biological and environmental systems.

In addition to pH and ionic strength, the influence of solvent environment on the fluorescence behavior of the CQDs was investigated. The highest fluorescence intensity of our CQDs was observed in water (Fig. 12c) This agrees with previous reports, where the strong polarization of water compared to organic solvents was shown to enhance dispersibility and stabilize surface states, thereby leading to the highest luminescence intensity^37,38^.

### Applications of the synthesized CQDs

#### Fluorescent ink

**Fig. 13:**
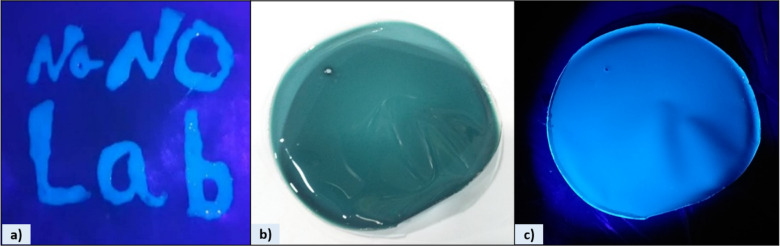
(**a**) Handwritten “Nano Lab” using CQDs ink, glowing blue under UV light (365 nm); (**b**) CQDs/PVA film under daylight; and (**c**) the same film under UV light (365 nm), exhibiting bright blue fluorescence.

Owing to their strong emission and good dispersibility, the aqueous CQDs dispersion could be directly utilized as a fluorescent ink for writing or printing. As demonstrated in (Fig. 13a), the handwritten letters “Nano Lab” on commercial paper using the CQDs solution emitted intense blue fluorescence under UV light (365 nm). This simple demonstration highlights the practical potential of the synthesized CQDs for use in anti-counterfeiting, security labeling, and optical display applications^39,40^.

### CQDs/PVA composite film

A uniform green CQDs/PVA film was successfully fabricated using the solution-casting method. As shown in (Fig. 13b, c), the film exhibited a smooth surface and homogeneous green color under daylight, indicating good dispersion of CQDs within the PVA matrix. When exposed to UV light (365 nm), the film emitted a bright blue fluorescence, demonstrating that the CQDs retained their strong fluorescent properties even in the solid state. The flexible nature of the composite film and its stable emission suggest strong interaction between CQDs and the hydroxyl groups of PVA, which help prevents aggregation and enhances fluorescence stability^41^. This behavior is consistent with the intense blue fluorescence observed for the CQDs in aqueous solution, further confirming the stability of their optical properties. These features indicate that the CQDs/PVA composite film holds promise for flexible optical applications and anti-counterfeiting materials^42,43^. Moreover, the preservation of strong solid-state fluorescence together with mechanical flexibility suggests potential applicability in stimulus-responsive optical devices, such as pH-sensitive indicators, humidity sensors, or mechanically responsive luminescent materials.

### Detection of Fe^3+^ ions

#### pH-dependent fluorescence quenching of Fe^3+^

**Fig. 14:**
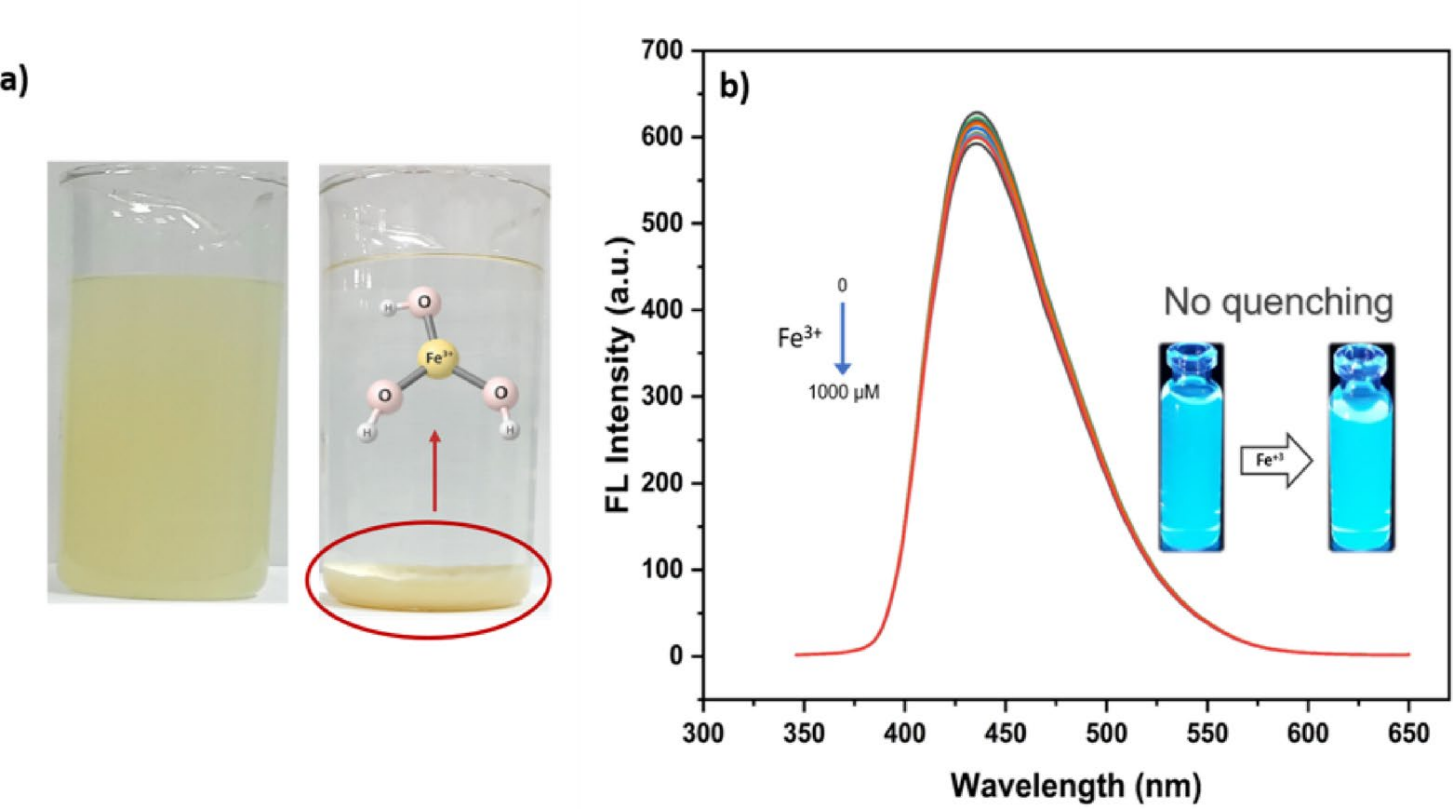
Figure X. pH-dependent behavior of Fe^3+^ at pH 8 (0.02 M PBS buffer). (**a**) Photographs showing the immediate formation of yellowish Fe(OH)_3_ precipitates upon preparation of Fe^3+^ solution (left) and the precipitate after sedimentation (right), (**b**) Fluorescence spectra of CQDs after addition of Fe^3+^ supernatant (0–1000 µM); the inset shows the CQDs dispersion before and after Fe^3+^ addition under 365 nm UV illumination.

Given that Fe^3+^ is one of the most widely studied quenchers for CQDs, its fluorescence quenching is known to be strongly influenced by solution pH. Therefore, systematic experiments were conducted to investigate the pH-dependent interaction between Fe^3+^ ions and the surface states of the prepared CQDs. At pH 8 (0.02 M PBS buffer), the preparation of Fe^3+^ solutions resulted in immediate turbidity due to the formation of insoluble Fe(OH)_3_ precipitates (Fig. 14a). To avoid artifactual effects from turbidity and light scattering, defined volumes of the clear supernatant were collected after the precipitate had settled and subsequently mixed with the CQDs solution, which was also prepared in the same buffer at pH 8. Fluorescence spectra of the CQDs were then recorded under these conditions. As shown in (Fig. 14b), the emission intensity exhibited negligible changes even at 1000 µM Fe^3+^, indicating that under alkaline conditions Fe^3+^ undergoes extensive hydrolysis and precipitation, leaving only a very low concentration of free ions available to interact with the CQDs^44,45^. Consequently, the apparent quenching observed at pH 8 originates from turbidity and light scattering, whereas no genuine quenching occurs in the clear supernatant solution.

**Fig. 15:**
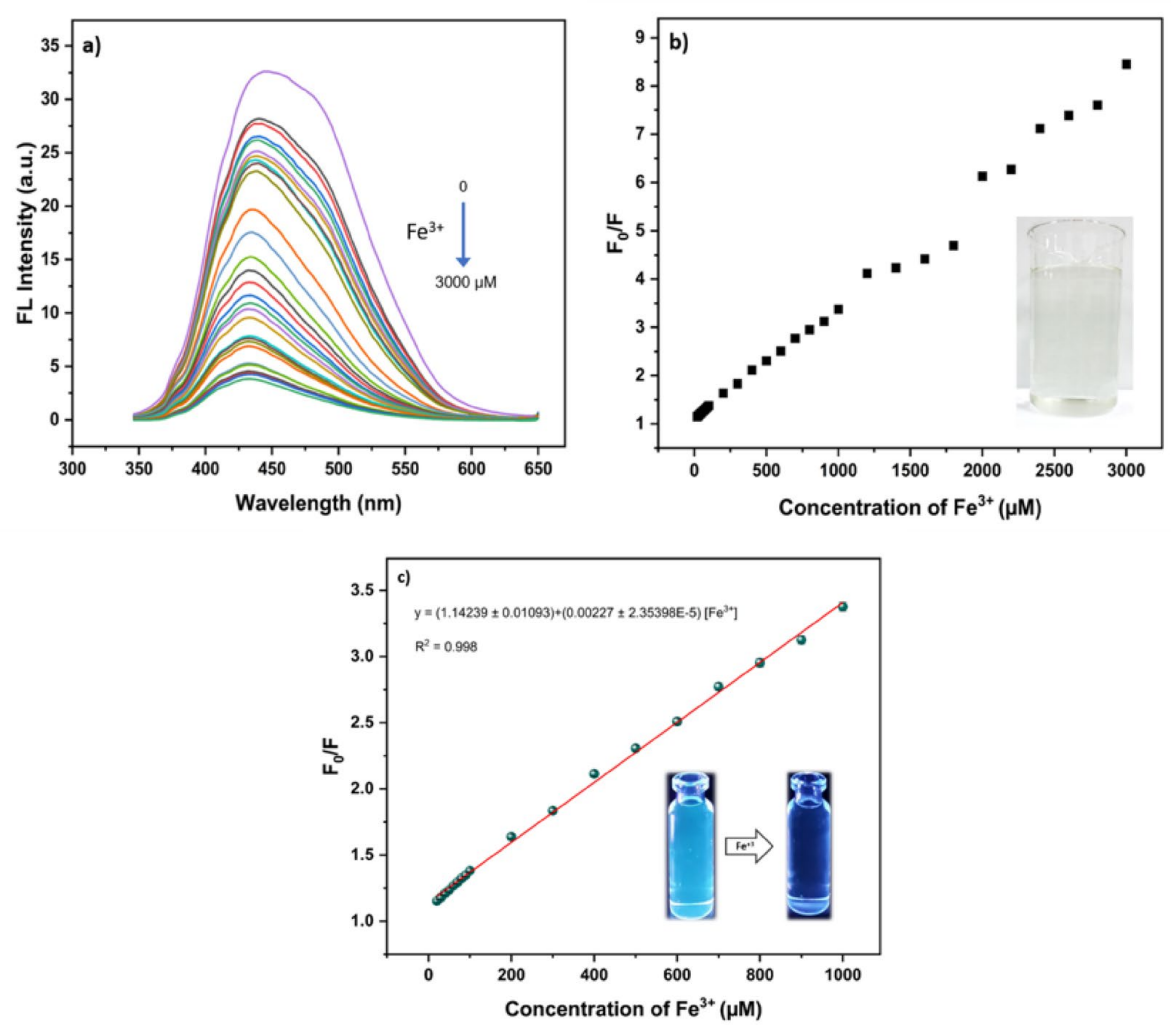
Fe^3+^induced fluorescence quenching of CQDs under acidic conditions (pH = 2). (**a**) Fluorescence emission spectra of CQDs upon successive addition of Fe^3+^ (0–3000 µM), (**b**) Stern–Volmer plot (F_0_/F) as a function of Fe^3+^ concentration; the inset shows the clear Fe^3+^ solution under acidic conditions, (**c**) Linear Stern–Volmer response in the concentration range of 20–1000 µM (R^2^ = 0.998); the inset shows the CQDs solution before and after Fe^3+^ addition under 365 nm UV light illumination.

To gain further insight into the role of pH in the quenching process, additional experiments were conducted under strongly acidic conditions (pH 2). At this pH, the fluorescence spectra of the CQDs exhibited a gradual decrease in emission intensity upon successive additions of Fe^3+^ ions over the concentration range of 0–3000 µM (Fig. 15a). This behavior suggests effective interaction between Fe^3+^ ions and the CQD surface. The Stern–Volmer analysis revealed a clear linear relationship between F_0_/F and Fe^3+^ concentration in the range of 20–1000 µM, with a good correlation coefficient (R^2^ = 0.998) (Fig. 15c). Based on this calibration, the limit of detection (LOD) and limit of quantification (LOQ) were determined to be 10.25 µM and 34.2 µM, respectively, using the standard formulas LOD = 3σ/s and LOQ = 10σ/s, where σ represents the standard deviation of blank signals (*n* = 5) and s the slope of the calibration curve. It is noteworthy that the LOD obtained in this work is higher than that reported in a previous study, which achieved an extremely low LOD of 0.14 µM at pH = 3. This discrepancy mainly arises from the experimental pH: while their CQDs exhibited maximum fluorescence at pH = 3 ^46^, our materials reached their highest intensity at pH = 8. However, at alkaline pH, Fe^3+^ readily hydrolyzes and precipitates as Fe(OH)_3_, generating turbidity artifacts that obscure genuine quenching effects. Therefore, the quenching measurements in this study were deliberately conducted at pH 2, where Fe^3+^ remains fully soluble and the observed decrease in fluorescence intensity genuinely reflects Fe^3+^–CQDs interactions.

To further elucidate the Fe^3+^-induced fluorescence quenching mechanism, possible interaction pathways were considered in light of previous reports on CQD-based sensing systems. In general, fluorescence quenching in carbon quantum dots may arise from inner filter effects (IFE), dynamic (collisional) quenching, static quenching via ground-state complex formation, or photoinduced electron transfer (PET)^47,48^. FTIR analysis confirmed the presence of oxygen- and nitrogen-containing functional groups, including –COOH and –NH₂ moieties, on the surface of the prepared N-CQDs. Owing to its high charge density and strong Lewis acidity, Fe^3+^ can interact with oxygen donor atoms of carboxyl groups, while nitrogen atoms may also participate through lone-pair donation. Such surface interactions have been widely reported to modulate the surface electronic environment of CQDs and contribute to fluorescence suppression^49,50^. Although –COOH and –NH₂ groups are largely protonated at this pH, partial interaction through oxygen donor sites remains feasible due to the strong Lewis acidity of Fe^3+^. A definitive distinction between static and dynamic quenching mechanisms would require time-resolved fluorescence measurements. Based on the present steady-state fluorescence results and relevant literature reports, the quenching behavior can be reasonably interpreted in terms of surface-related interactions under acidic conditions. Furthermore, many previous reports did not explicitly mention the pH conditions under which Fe^3+^ quenching experiments were performed or carried them out near neutrality, despite the fact that Fe^3+^ speciation and precipitation are highly pH-dependent^32,51–53^. By explicitly addressing this aspect, our study emphasizes the importance of selecting suitable solution conditions to ensure reliable and chemically meaningful quenching data. A comparison with reported CQD-based Fe^3+^ sensors is summarized in Table 1. Although the detection limit of the proposed sensing system is higher than that reported for some nM-level CQD-based Fe^3+^ sensors, the present method offers a wide linear detection range, good environmental stability, and reliable performance under strongly acidic and high-ionic-strength conditions. These features make the system particularly suitable for monitoring Fe^3+^ at moderate to high concentration levels, such as those commonly encountered in industrial wastewater and other high-ionic-strength aqueous environments.

**Table 1:** Comparison of CQD-based fluorescent sensors for Fe^3+^ detection.

Probe	Materials	Linear range (µM)	Limit detection (LOD)	Test pH values	References
N-CQDs	Citric acid and ethylenediamine	0–250 ;250–1200	1.68 µM	–	^54^
CQDs	zinc gluconate	0–200	1.9 µM	–	^55^
CQDs	Poria cocos	0–750	1.98×$\:{10}^{-3}$ nM	–	^56^
B, N-CQDs	trisodium citrate, urea and boric acid	0–100	107 nM	5	^57^
CQDs	cranberry beans	30–600	9.55 µM	5.10	^58^
N-CQDs	Diammonium hydrogen citrate and urea	0–300	19 µM	–	^59^
N-CQDs	L-glutamic acid and anhydrous ethylenediamine	8–80	3.8 µM	7	^60^
N-CQDs	Ethylenediamine and phytic acid	1–10 ;10 -1000	0.39 µM	7.40	^52^
N-CQDs	CMC and LPEI	1-400	0.14 µM	3	^46^
N-CQDs	Alginic acid and ethanediamine	0–50	10.98 µM	4	^61^
N-CQDs	Citric acid and urea	20–1000	10.25 µM	2	^This work^

### Selectivity and interference studies toward Fe^3+^

**Fig. 16:**
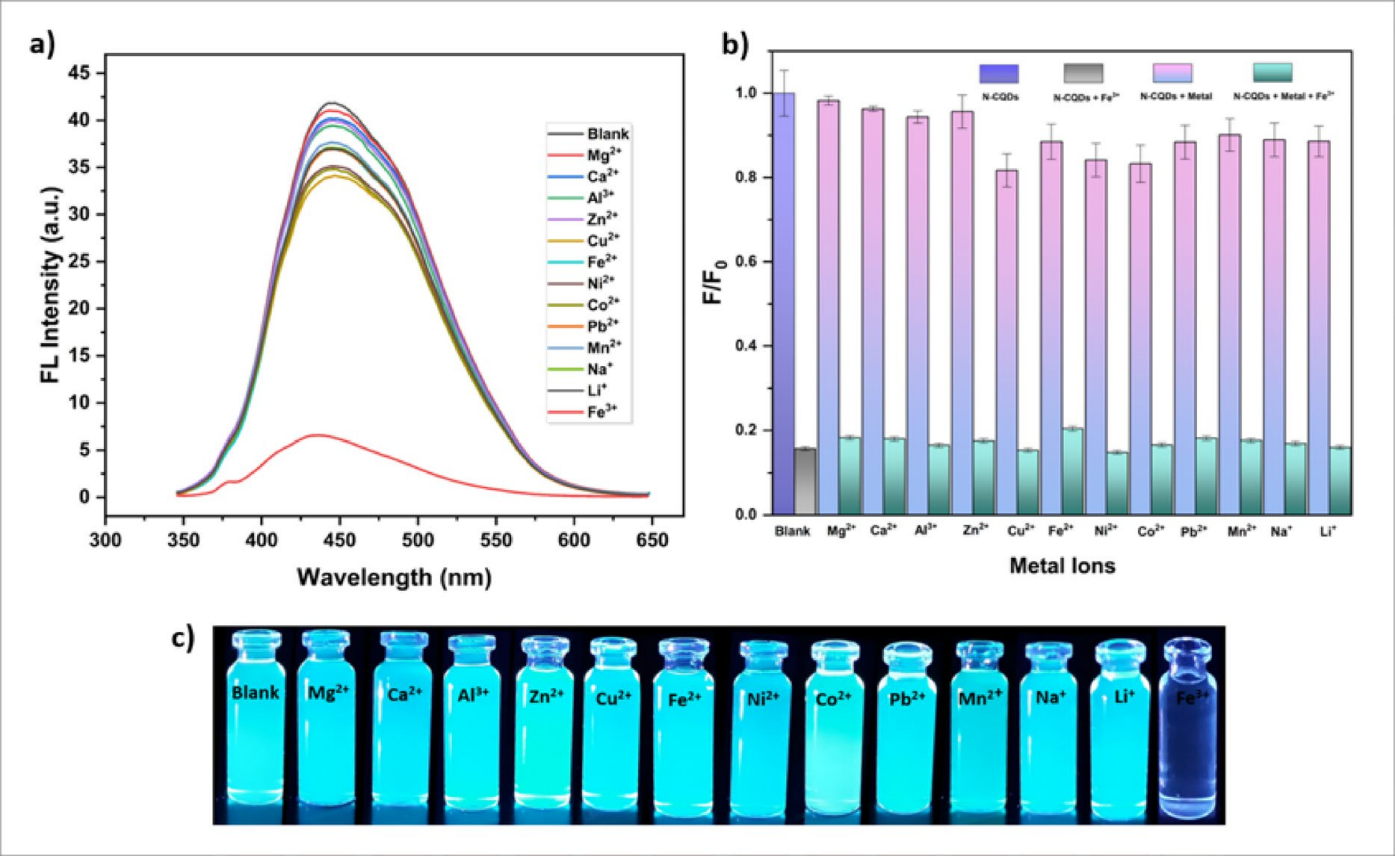
(**a**) Fluorescence emission spectra of N-CQDs in the presence of various metal ions (1 mM, pH 2), (**b**) Relative fluorescence response (F/F_0_) of N-CQDs toward individual metal ions and mixtures of each metal ion with Fe^3+^ under identical conditions, (**c**) Photographs of N-CQD solutions containing different metal ions under UV irradiation (365 nm), highlighting the pronounced quenching effect of Fe^3+^.

In order to evaluate the specificity and sensitivity of the prepared N-CQDs toward Fe^3+^ ions, the fluorescence responses of the N-CQDs in the presence of various metal ions were systematically investigated under identical experimental conditions (pH = 2). A series of common metal ions, including Mg^2+^, Ca^2+^, Al^3+^, Zn^2+^, Cu^2+^, Fe^2+^, Ni^2+^, Co^2+^, Pb^2+^, Mn^2+^, Na⁺, and Li⁺, were individually added to the aqueous N-CQD solution at an identical concentration (1 mM), and the corresponding fluorescence intensities were recorded. As shown in (Fig. 16a), the addition of Fe^3+^ resulted in a pronounced decrease in fluorescence intensity, whereas other metal ions induced only negligible changes. This observation indicates that the fluorescence quenching efficiency of Fe^3+^ is significantly higher than that of the other tested metal ions, demonstrating the high selectivity of the CQDs toward Fe^3+^. To further assess the anti-interference capability of the sensing system, competitive experiments were carried out by introducing Fe^3+^ ions into N-CQD solutions containing coexisting metal ions. As illustrated in (Fig. 16b), the fluorescence quenching induced by Fe^3+^ remained essentially unchanged in the presence of these potentially interfering species, confirming the strong anti-interference capability of the proposed sensing system.

### Analysis of real water samples

In addition to selectivity, the practical applicability of the proposed sensing system was validated through spike–recovery experiments using drinking water samples. Known amounts of Fe^3+^ were spiked into the water samples, and the fluorescence responses were analyzed based on the established calibration curve. As summarized in Table 2, the recoveries ranged from 97.88% to 101.79%, with relative standard deviation (RSD) values below 1.3% (*n* = 3). These results demonstrate good accuracy and repeatability of the proposed method in real water matrices, confirming its suitability for monitoring Fe^3+^ in environmental water samples.

**Table 2:** Determination of Fe^3+^ in drinking water samples by spike–recovery experiments, showing recoveries and relative standard deviation (RSD, *n* = 3).

Spiked (µM)	Detected (µM)	Recovery (%)	RSD (%, *n* = 3)
200	197.5	99.10	0.79
350	347.18	98.35	0.76
500	496.67	97.88	1.28
700	700.64	101.79	0.72
900	900.28	100.53	1.15

## Methods

### Synthesis of N-doped CQDs

The CQDs were synthesized via a one-step hydrothermal method using citric acid as the carbon precursor and urea as the nitrogen precursor. Briefly, 1.0 g of citric acid and 1.5 g of urea were dissolved in 25 mL of deionized water under magnetic stirring until a clear solution was obtained. The solution was then transferred into a Teflon-lined autoclave and heated at 180 °C for 4 h. After natural cooling to room temperature, the product was centrifuged at 10,000 rpm for 15 min to remove carbonized residues, and the supernatant was vacuum-filtered through a 0.22 μm membrane. To remove residual salts and secondary by-products, the aqueous CQD dispersion was mixed with methanol at a volume ratio of 1:2 (CQDs: MeOH). The mixture was gently agitated and centrifuged at 9,000 rpm for 10 min. The supernatant containing the purified CQDs was carefully collected, while the precipitated impurities were discarded. This MeOH-assisted purification procedure was repeated three times to remove residual impurities.

### Preparation of CQDs/PVA fluorescent film

Polyvinyl alcohol (PVA, 10 wt%) was dissolved in deionized water at 80 °C with stirring until a clear solution was obtained. A CQDs solution was then added to the PVA solution and gently stirred thoroughly to ensure homogeneity. The resulting mixture was poured into a Petri dish mold and left to dry at room temperature for 24 h to allow slow water evaporation and polymer film formation. The obtained film was green in color, flexible, and easy to peel from the mold.

#### Detection of Fe^3+^ ions

A stock Fe^3+^ solution (4 mM) was prepared by dissolving FeCl_3_ in a pH-2 nitric acid medium. A series of Fe^3+^ solutions with final concentrations ranging from 20 to 3000 µM were then obtained by appropriate dilution with the same acidic medium. For fluorescence measurements, 1 mL of the CQDs solution (0.1 g/L) was transferred into a 10 mL volumetric flask, yielding a final CQDs concentration of 0.01 g/L after dilution to the mark. Aliquots of Fe^3+^ solutions were subsequently added to obtain the desired final Fe^3+^ concentrations within the same 10 mL system. The samples were kept at room temperature for 10 min before recording the fluorescence spectra. Fluorescence measurements were performed at an excitation wavelength of 335 nm with emission monitored at 435 nm. The calibration curve was deduced based on the relationship between Fe^3+^ concentration and fluorescence quenching efficiency F_0_/F, where F_0_​ and F represent the fluorescence intensities of CQDs in the absence and presence of Fe^3+^, respectively.

#### Characterization of CQDs

Atomic force microscopy (AFM) images were recorded using an AFM (Nanosurf, Switzerland, easyScan 2 operated in tapping mode), to analyze particle surface topography and height distribution. High-resolution transmission electron microscopy (HRTEM) images and selected-area electron diffraction (SAED) patterns were acquired using HRTEM, (Philips CM200, Netherlands) operated at (200KV), providing insight into particle morphology, lattice fringes, and crystallinity. Image processing and quantitative measurements of HRTEM micrographs were performed using ImageJ software (NIH, USA). Dynamic light scattering (DLS) and zeta-potential measurements were performed using a Malvern Zetasizer Nano ZS (Malvern Instruments, UK) to determine hydrodynamic particle size distribution and colloidal stability. Elemental composition was analyzed via energy-dispersive X-ray spectroscopy (Oxford Instruments X-Max 80, UK). X-ray diffraction (XRD).

patterns were obtained using a PANalytical X’Pert PRO diffractometer (Netherlands) equipped with Cu Kα radiation (λ = 1.5406 Å) over a 2θ range of 9°–80° to examine structural crystallinity. Raman spectra were recorded using a LabRAM HR Evolution spectrometer (Horiba, France) with a 632 nm excitation laser. Fourier-transform infrared spectra (FTIR) were collected using an IR Affinity-1 S spectrometer (Shimadzu, Japan) within the 4000–400 cm^−1^ range. UV–visible absorption spectra were measured using a Cary 5000 UV–Vis–NIR spectrophotometer (Agilent Technologies, USA) over the wavelength range of 200–800 nm, and photoluminescence measurements were acquired on a JASCO FP-2600 fluorescence spectrometer (Japan).

#### Quantum yield measurements

The quantum yield (QY) of the CQDs was determined using the relative slope method, following previously reported procedures^62^. Quinine sulfate (QY = 0.54 at 360 nm) in 0.1 M H₂SO₄ (refractive index η = 1.33) was used as the reference standard for comparison with the aqueous CQDs solution (η = 1.33). To minimize inner-filter and re-absorption effects, the absorbance of all solutions at the excitation wavelength was kept below 0.1. Absorbance spectra were recorded using a UV–Vis spectrophotometer, and emission spectra were acquired at an excitation wavelength of 360 nm. For both the sample and the standard, the integrated emission intensity was plotted as a function of absorbance, and the slopes extracted from the corresponding linear fits were used to compute the QY according to:

$$\:{\mathrm{Q}}_{\mathrm{x}}={\mathrm{Q}}_{\mathrm{s}\mathrm{t}}({m}_{\mathrm{x}}/{m}_{st}){({{\upeta\:}}_{\mathrm{x}}/{{\upeta\:}}_{\mathrm{s}\mathrm{t}})}^{2}$$

where $\:{\mathrm{Q}}_{\mathrm{s}\mathrm{t}}\:$is the QY of the reference standard, m denotes the slope of the integrated-intensity versus absorbance plot, and η represents the refractive index of the solvent. The subscript “st” refers to the quinine sulfate and “x” refers CQDS. Since both reference and sample were measured in aqueous media, $\:{{\upeta\:}}_{\mathrm{x}}/{{\upeta\:}}_{\mathrm{s}\mathrm{t}}=1$. All measurements were performed at room temperature.

## Conclusion

In summary, nitrogen-doped CQDs were successfully synthesized via a facile hydrothermal approach using citric acid and urea, yielding highly fluorescent, water-dispersible nanomaterials with a quantum yield of 37.8%. Comprehensive structural, morphological, and spectroscopic analyses confirmed their nanoscale dimensions, partially graphitic structure, and rich surface functionalization. The CQDs exhibited excellent fluorescence stability over a broad range of pH values, high ionic strength, and different solvent environments, underscoring their robustness for practical applications. A systematic investigation of Fe^3+^ sensing revealed that alkaline conditions lead to Fe(OH)_3_ precipitation and unreliable quenching behavior, whereas strongly acidic conditions ensure full Fe^3+^ solubility and genuine fluorescence quenching. Under acidic conditions (pH 2), the CQDs enabled reliable Fe^3+^ detection with a broad linear range and satisfactory sensitivity. Furthermore, the successful fabrication of fluorescent ink and CQDs/PVA composite films highlights the versatility of the synthesized CQDs for optical and anti-counterfeiting applications. Overall, this study provides both fundamental insight and practical guidance for CQD-based sensing systems, emphasizing the importance of carefully controlled chemical environments for accurate fluorescence-based detection.

## Data Availability

The datasets generated and/or analyzed during the current study are available from the corresponding author on reasonable request.
